# MiR-3571 modulates the proliferation and migration of vascular smooth muscle cells by targeting claudin 1

**DOI:** 10.7150/ijms.64639

**Published:** 2022-03-06

**Authors:** Yilin Xie, Juanjuan Tan, Yingchun Qin, Yong Cao, Yicheng Wang, Aihua Li, Zhaoxia Wang, Zhongdong Qiao, Zhiqiang Yan

**Affiliations:** 1 Shanghai Jiao Tong University - Minhang Campus, School of Life Science and Biotechnology, Shanghai Key Laboratory for Reproductive Medicine, Shanghai, China; 2 Shanghai Jiao Tong University - Minhang Campus, School of Chemistry and Chemical Engineering, State Key Laboratory of Metal Matrix Composite Materials and Shanghai Key Lab of Electrical Insulation and Thermal Ageing, Shanghai, China; 3 Shanghai University of Traditional Chinese Medicine, Shanghai, China; 4 Shanghai Jiao Tong University School of Life Sciences and Biotechnology, Shanghai, China; 5 Anhui University of Science and Technology, Huainan, Anhui, China; 6 Shanghai Jiao Tong University Laboratory Animal Center, Shanghai, China; 7 Shanghai Jiao Tong University affiliated sixth people's hospital south campus, Central Laboratory, Shanghai, China

**Keywords:** DNA hydroxymethylation, miR-3571, Claudin-1, Vascular smooth muscle cell, Hypertension

## Abstract

**Background and aims:** The miRNA-based post-transcription modification has been extensively studied in hypertension. It however remains elusive how miRNA expression is regulated in this pathological process. We hypothesize that hydroxymethylation in the promoter regions tightly controls the levels of key miRNAs, which in turn affects the development of hypertension.

**Methods:** The levels of hydroxymethylation in the promoter regions from thoracic aortic tissues were compared between spontaneously hypertensive rats (SHRs) and normotensive Wistar-Kyoto rats (WKYs), using hydroxymethylcytosine DNA immunoprecipitation (hMeDIP) sequencing. The altered hydroxymethylation level of miR-3571 was confirmed by glucosylation-coupled hydroxymethylation-sensitive qPCR. We further identified claudin 1(CLDN1) as a key target of miR-3571 via bioinformatic prediction (targetscan) and dual-luciferase activity assays. Finally, we analyzed the contribution of miR-3571/CLDN1 axis in the proliferation and migration of vascular smooth muscle cells (VSMCs).

**Results:** The hydroxymethylation level of miR-3571 promoter region in thoracic aortic tissue from spontaneously hypertensive rats was lower than that from normotensive Wistar-Kyoto rats. Accordingly, the expression of miR-3571 was lower during hypertension, with up-regulated CLDN1 protein levels. More importantly, we found that miR3571 overexpression led to phenotypic changes of VSMCs, and inhibited the proliferation and migration of muscle cells via suppressing CLDN1 as well. Our findings further suggested that CLDN1 up-regulation increase the activity of ERK1/2 in VSMCs.

**Conclusions:** Our study suggested that hydroxymethylation in the promoter regions controlled the level of miR-3571 and revealed the important roles of miR-3571 and CLDN1 in VSMCs during the development of hypertension. In addition, our results also indicated that miR-3571/CLDN1 axis regulated the functions of VSMCs via the ERK1/2 pathway. Taken together, our findings support miR-3571 as a novel biomarker for the diagnosis and prevention of hypertension.

## Introduction

Hypertension is considered a major risk factor for stroke, and cardiovascular and kidney disease. The principal pathological changes in hypertension are vascular remodeling, which manifests as a thickening of the medial layer of the aorta 1. As demonstrated in multiple studies, the hyper-mechanical strain experienced by the arterial walls during hypertension and the continuous increase in cyclic strain (CS) is strongly related to vascular remodeling through the regulation of VSMC function 2-5. The principal pathological feature of vascular remodeling is phenotypic modulation of VSMCs, from contractile to dedifferentiated, resulting in aberrant proliferation, migration, and extracellular matrix secretion 1, 6, 7. Unlike the majority of mature cells, smooth muscle cells (SMCs) are remarkably plastic and can dedifferentiate in response to environmental cues, demonstrating the complexity of gene expression regulation 7-9. Therefore, aberrant proliferation and migration of VSMCs and the expression of marker proteins are important indicators of vascular remodeling.

MiRNA-based post-transcription modification mechanisms controlled by small noncoding micro-RNAs (miRNAs) contribute to the regulation and pathogenesis of hypertension. As reported in multiple studies, miR-145-5p, miR-96, miR-130, miR-26a, and many other miRNAs have been reported to participate in the regulation and function of VSMCs in hypertension 10-12. It however remains elusive how miRNA expression is regulated in this pathological process. Methylation of promoter CpG-islands is common to both protein-coding genes and miRNA genes and has been reported many times to regulate miRNA expression in cancer 13-15. What's more, Krishnan et al. reported that miR-510 was upregulated in blood samples from hypertension patients and hypermethylation is corroborated with miR-510 expression in blood samples 16. DNA methyltransferases (DNMTs) catalyze the fifth carbon atom of cytosine to form 5-methylcytosine (5-mC) which ultimately leads to gene silencing to inhibit transcription while DNA demethylation occurs via 5-mC oxidation catalyzed by a family of protein called Ten-eleven translocation (TET) enzyme which usually leads to DNA demethylation and active gene expression 17. Interestingly, a recent study suggests that TET2 is downregulated in atherosclerotic lesions and participates in pathophysiological progression of atherosclerosis 18. In addition, Liu et al. reported that the loss of TET2 significantly suppressed the enrichment of 5-hydroxymethyl-cytosine (5-hmC) in the promoter of smooth muscle-myosin heavy chain (MYH11) and smooth muscle actin (ACTA2) and resulting in the modulation of VSMC phenotype from contractile to dedifferentiated and vascular remodeling 19. The latest research revealed that decreased expression of the epigenetic regulator TET2 is associated with spontaneous development of pulmonary hypertension and has potential as a biomarker for pulmonary arterial hypertension (PAH) 20. Although DNA methylation is an important mechanism for miRNA regulation, DNA hydroxymethylation has not been explored in miRNA regulation. Thus, in current study, we explored hydroxymethylcytosine DNA immunoprecipitation (hMeDIP) sequencing in the tissue of the thoracic aortas of SHRs and WKYs, finding that hydroxymethylation in the promoter region of miR-3571 in the thoracic aorta was different in SHRs than in WKYs. A recent report demonstrated that downregulation of miR-3571 was associated with cardiac dysfunction in rats with selenium deficiency 21. Therefore, miR-3571 may be an appropriate target for studying DNA hydroxymethylation in miRNA regulation.

Accordingly, differential hydroxymethylation of the promoter region of miRNA-3571 was found in thoracic aortic tissues from WKYs and SHRs using hMeDIP-Seq and verified in the present study by glucosylation-coupled hydroxymethylation-sensitive qPCR. Furthermore, the effect of miR-3571 and its possible target gene on the proliferation and migration of VSMCs was investigated *in vitro* and the possible underlying mechanisms were explored. Together, the results may provide new insight into the pathogenesis of hypertension.

## Materials and methods

### 2.1 Animals

SHRs and WKYs were purchased from Beijing Vital River Laboratory Animal Technology Company (Beijing, China). Sprague-Dawley rats (SDRs) were obtained from Shanghai Laboratory Animal Research Center (Shanghai, China). All experimental protocols were approved by the Institutional Animal Care and Use Committee of Shanghai Jiao Tong University (Shanghai, China) and the Ethics Committees of Shanghai Jiao Tong University. All experiments were conducted in accordance with the relevant guidelines and regulations for the Care and Use of Laboratory Animals. A detailed description of the SHRs and WKYs used for sequencing is provided in Supplementary Table 1 and Supplementary Figure S3.

### 2.2 Hydroxymethylcytosine DNA immunoprecipitation (hMeDIP) sequencing and analysis

hMeDIP-Seq was performed by CloudSeq Biotech Inc. (Shanghai, China). Briefly, genomic DNA was extracted using a DNeasy kit (Qiagen Inc., Germany) and sonicated into 100-300 bp fragments. Adaptors were then ligated to the genomic DNA fragments in accordance with the Illumina protocol (Illumina, Inc., USA). The ligated DNA fragments were immunoprecipitated with 5-hydroxymethylcytosine (5hmC) antibodies (Active Motif) overnight at 4°C. The DNA-antibody mixture was incubated with protein G Dynabeads (Life Technologies) for 2 h at 4°C and washed three times with immunoprecipitation buffer. The beads were then treated with proteinase K for at least 3 h at 55°C, and the immunoprecipitated DNA was purified using phenol-chloroform extraction followed by ethanol precipitation. IP DNA and input DNA were amplified by PCR using Illumina primers, and then purified to obtain DNA libraries. Next-generation sequencing was performed on the pooled libraries using an Illumina Hiseq instrument with 150bp paired-end reads. The raw data generated after sequencing, underwentbase calling and quality filtering on an Illumina HiSeq4000 sequencer. Firstly, only data satisfying Q30 quality control standards were retained. After adaptor-trimming and removal of low-quality reads using cut adapt (v1.9.1) software, high quality reads (clean reads) were generated. These clean reads were aligned to the rat genome (ucsc rn6) using the default parameters of BOWTIE software (V2.1.0). Peak calling was performed using MACS1.4 software. Differentially hydromethylated regions (DhMRs) were identified using diffReps software. The enriched peaks and DhMRs were then annotated using the latest UCSC RefSeq database that connected peak information with gene annotation. The enriched peaks were then visualized using the UCSC genome browser.

### 2.3. Cell culture and transfection

Primary culture of VSMCs from the thoracic aortas of SD rats was initiated by explant culture. Primary VSMCs were cultured in Dulbecco's modified Eagle medium (DMEM) supplemented with 10% fetal bovine serum (FBS; ThermoFisher, USA) at 37°C in an atmosphere containing 5% CO_2_. The purity of the VSMCs was confirmed by the analysis of smooth muscle α-actin (SMA). Cells from passages 3-7 were used in all experiments. Prior to transfection, VSMCs were seeded in 6well plates (2 ×10^6^ cells in each well) then cultured until 50-60% confluent. VSMCs were then transfected with miR-3571 mimic (40 nM), miR-3571 antagomirs (40 nM) or CLDN1 siRNA (40 nM) (Table 5) and their corresponding negative control(40 nM) using siRNA-mate (GenePharma, China). All transfections were performed in accordance with the manufacturer's instructions. After an incubation period of 24 h or 48 h, total mRNA and protein were analyzed using RT-qPCR and Western blotting.

### 2.4 Application of Mechanical Stretch

For the application of mechanical cyclic stretch, VSMCs were plated on type I collagen-coated flexible silicone-bottomed plates (Flexercell International, USA) and then were subjected to CS. VSMCs were subjected to mechanical stress by 5% or 15% elongation of elastomer-bottomed plates for a default time program (12 h or 24 h) by a Flexcell® FX-5000™ Tension System (Flexcell International, USA).

### 2.5 Glucosylation-Coupled Hydroxymethylation-Sensitive qPCR

The level of hydroxymethylation within the miR-3571 promoter region was measured using a Quest 5-hmC detection kit (ZymoResearch, USA) in accordance with the manufacturer's instructions. A HaeⅢ restriction enzyme was used to digest the modified DNA. The sequence of the primer used is displayed in Table 1.

### 2.6 RT-qPCR

TRIzol reagent (Invitrogen, USA) was used to extract total RNA inaccordance with the manufacturer's instructions which was then reverse transcribed into cDNA using an NEB reverse transcription kit (Thermo Fisher Scientific, USA). qPCR was performed with a 2 × SYBR Green master mix (Takara, Japan) using a 7500 Real-Time PCR System (Applied Biosystems). The qPCR primer sequences are listed in Table 2. Changes in gene expression in both cells and tissue samples were calculated using the 2^ -ΔΔCt^ method.

### 2.7 Dual-Luciferase Activity Assay

The target genes for miR-3571 were predicted using the TargetScan database, version 7.1 (http://www.targetscan.org/vert_71/). A luciferase assay was used to measure the targeted association between miR-3571 and the 3'-UTR of CLDN1. Briefly, wildtype (WT) and mutanttype (MUT) CLDN1 3' UTR pmirGLO luciferase reporter vectors and their specific primers were designed, as detailed in Table 3. MiR-3571 mimics or miR-3571 NC and each WT or MUT luciferase reporter vector were cotransfected into human embryonic kidney (HEK) 293T cells using Lipofectamine 3000 (Invitrogen, USA). Luciferase activity was measured using a dual luciferase assay kit (YPHbio, China) after 24 h of transfection. Specific target activity was expressed as the relative ratio of firefly luciferase luminescence to that of renilla luciferase.

### 2.8 Lentiviral Vector Production and Transduction

Lentiviral CLDN1 and miR-3571 overexpression vectors for CLDN1 and miR-3571 overexpression and LV3-shNC were purchased from GenePharma (China). All lentiviral vectors were packaged in HEK293FT cells and produced as described previously 22. Stable cell lines were established by transfecting VSMCs with purified virus, after which stable batches of cells were selected using 3 µg/mL puromycin.

### 2.9 Western blot analysis

VSMCs were collected in RIPA buffer (Thermo Scientific, Rockford, IL, USA) containing a proteinase inhibitor cocktail (Roche, USA). Protein lysates (40 µg/lane) were loaded onto 10% SDS-PAGE gels and transferred onto nitrocellulose membranes. The membranes were blocked with 5% non-fat milk for 1 h then incubated with primary antibodies (1:1000 dilution) against glyceraldehyde 3-phosphate dehydrogenase (GAPDH; Sigma, St. Louis, MO, USA), MMP2, MMP9, CLDN1 (Abcam, UK), SM22 , calponin, ACTA2 (Bio Basic, Canada), PCNA (Bioworld, USA), beta-tubulin, MMP9 (Proteintech, USA) , p27, cyclin D1, cyclin E1, ERK, p-ERK, AKT, and p-AKT (Cell Signaling Technology) at 4°C overnight. After washing with TBST three times, membranes were incubated with a secondary antibody (1:5000 dilution) (Proteintech, USA). Finally, the membranes were visualized using enhanced chemiluminescence (ECL; Pierce, Rockford, USA) and quantified using ImageJ software (NIH, USA).

### 2.10 Cell proliferation assay

Cell proliferation was examined using an EdU assay kit purchased from Beyotime Biotechnology (China) and a cell counting kit-8 (CCK8) assay from MedChem Express (USA). Briefly, the three stable cell lines (LV-NC, OE-LV-miR-3571, and OE-LV-CLDN1) were trypsinized then seeded into the wells of 96-well micro titerplates in DMEM supplemented with 10% FBS at an initial concentration of 1 × 10^4^ cells per well and incubated at 37°C in an atmosphere containing 5% CO_2_ for 24 h. The medium was exchanged for DMEM supplemented with 1% FBS and the cells incubated for a further 24 h. The viability of the three stable cell lines was then examined in accordance with the manufacturer's instructions. The result of CCK8 assay were calculated from relative light absorbance while cells from the EdU assay were imaged using a fluorescence microscope (IX71 Olympus Corporation, Japan) and analyzed with image J software. Cell viability in EdU assay = numbers of cells with fluoresing red (stained with Azide 555)/ numbers of cells fluoresing blue (stained with Hoechst 33342).

### 2.11 Cell cycle analysis

The three stable cell lines (LV-NC, OE-LV-miR-3571, and OE-LV-CLDN1) were seeded in 6-well plates at a density of 2 × 10^5^ cells/well (Safar et al., 1981) and incubated in an atmosphere of air containing 5% CO_2_ at 37°C for 24 h. The culture medium was exchanged for DMEM supplemented with 1% FBS and the cells incubated for a further 24 h. After incubation, the VSMCs were harvested, re-suspended in 70% ethanol, and then incubated at 4°C for 24 h. The fixed cells were washed twice with PBS then incubated at 37°C in the dark for 30 min using a solution containing DNase-free RNAse (200 mg/mL) and propidium iodide (50 mg/mL). Finally, the cells were filtered once through a 400-mesh sieve prior to flow cytometric analysis using a FACSCanto II Flow Cytometer (BD Biosciences, USA).

### 2.12 Cell migration assay

Cell migration was examined in a wound healing assay, as described previously 22, where a scratch was created in a monolayer of cells and migration measured after 0 h, 12 h, and 24 h. A migration index was calculated using the formula:

mean V= (wound area at 0 h-wound area at 24 h)/wound area at 0 h × 100%

### 2.13 Statistical analysis

Comparisons between groups were analyzed using an unpaired t-test. Differences in three or more groups were evaluated using one-way analysis of variance (ANOVA). P-value<0.05 were considered significantly different. Data are expressed as means ± SD.

## Results

### 3.1 Identification of differentially hydroxymethylated promoter regions of miRNA in the thoracic aortas of SHRs and WKYs

Using hMeDIP-Seq, it was found that 14,535 hydroxymethylated regions displayed significant differential expression in thoracic aortic tissues from SHRs compared with WKYs, of which 8,990 regions were significantly up-regulated, and 5,545 regions were significantly down-regulated. Only 2.84% of the differentially hydroxymethylated regions were found to be located within the promoter region. Specifically, 250 promoter regions were more hydroxymethylated and 169 were less hydroxymethylated (Supplementary Figure S1). Finally, of these 419 differentially hydroxymethylated promoter regions, 5 miRNAs were identified in which the hydroxymethylated regions displayed significant differential expression in SHRs compared with WKYs. The 5 miRNAs are presented in Table 4.

### 3.2 Validation of the hydroxymethylated promoter regions in miR-3571 from SHRs and WKYs

**Figure 1:**
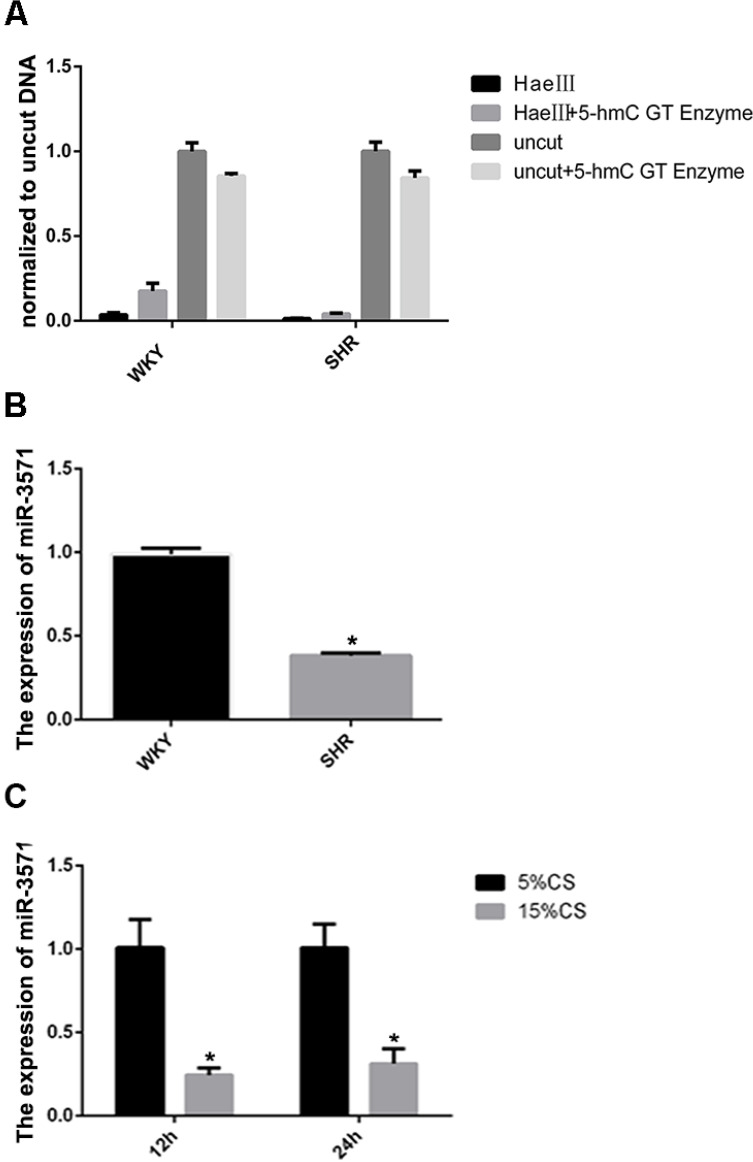
** MiR-3571 expression was lower in the aortas of SHRs than in WKYs while levels of hydroxymethylation in the miR-3571 promoter region of the aortas of SHRs were lower than those of WKYs. A.** The levels of hydroxymethylation in the miR-3571 promoter region of the aortas of SHRs and WKYs were measured by Glucosylation-Coupled Hydroxymethylation-Sensitivity qPCR. **B.** Expression of miR-3571 in the aortas of SHRs and WKYs were measured by RTqPCR. * p < 0.05 versus WKYs. C. Expression of miR-3571 in VSMCs subjected to 5% or 15% CS for 12h or 24h were measured by RT-qPCR. * p < 0.05 versus 5% CS. Data represent means ± SD of at least three separate experiments. Statistical comparisons were conducted using a Student's t test.

In an earlier hMeDIP sequencing study, we found that 5 miRNAs extracted from the thoracic aortas of SHRs had differential levels of hydroxymethylation in the promoter regions compared with WKYs. In the current study, an initial aim was to verify these analyses. Using glucosylation-coupled hydroxymethylation-sensitive qPCR, the level of hydroxymethylation within the miR-3571 promoter region was found to be lower in the aortas of SHRs than WKYs (Figure 1A), as was the expression of miR-3571, as measured with RT-qPCR (Figure 1B). Furthermore, the expression of miR-3571 was found to be down-regulated in VSMCs subjected to 15% CS compared with 5% CS (Figure 1C). And we have found that 5% CS have no significant effect on the expression of miR-3571 in VSMCs compared with baseline unstrained VSMCs (Supplementary Figure S4). Thus, it is possible to speculate from these results that miR-3571 may be involved in the progression of hypertension, and indicating that the function of miR-3571 warranted further investigation.

### 3.3 CLDN1 is a downstream target of miR-3571

**Figure 2:**
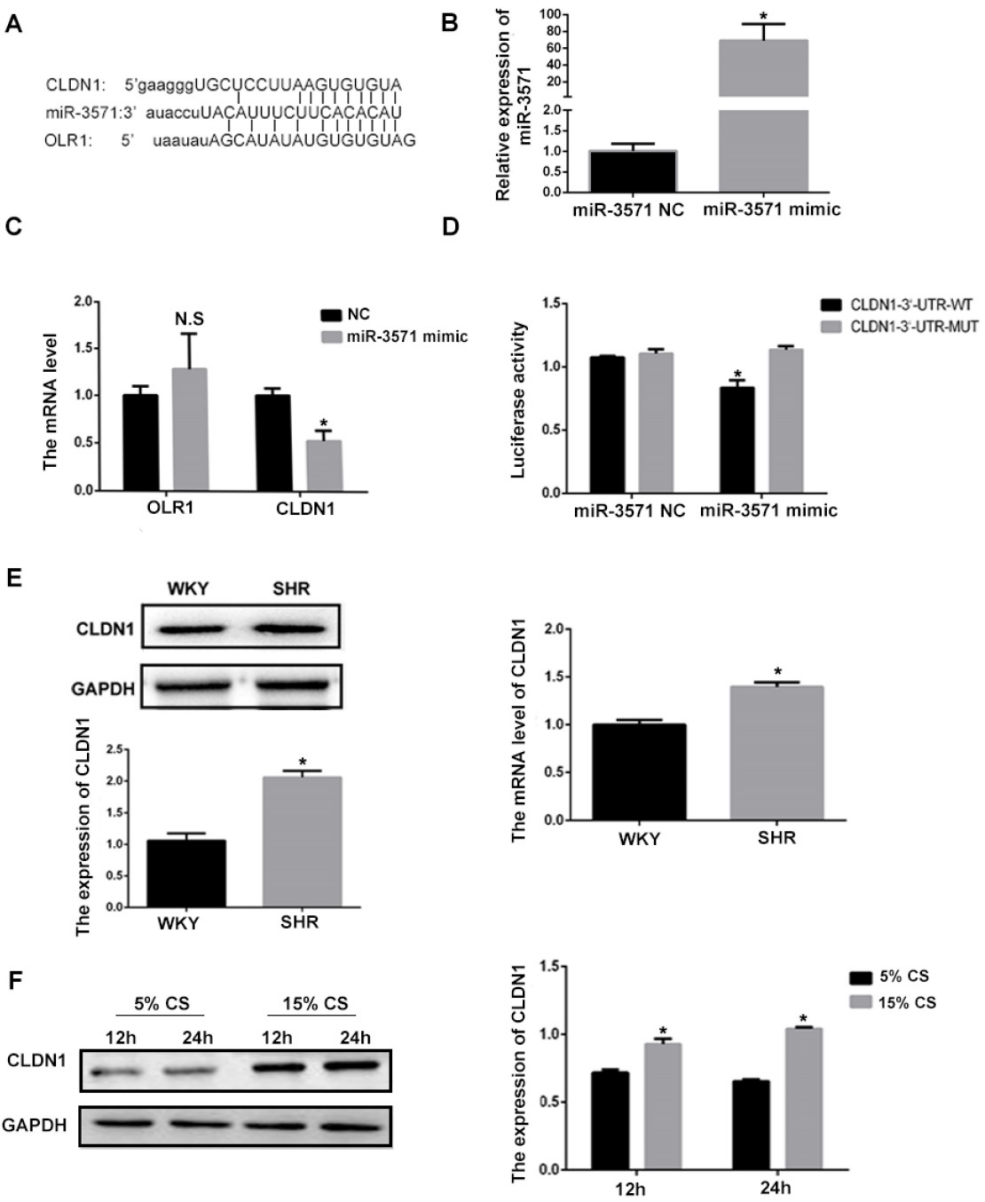
** CLDN1 is a downstream target of miR3571. A.** Predicted target binding region between miR3571, and CLDN1 and OLR1. **B.** Expression of miR3571 after transfection with miR3571 mimic or miR-3571 NC was measured by RTqPCR. *p < 0.05 versus miR-3571 NC. **C.** Expression of CLDN1 and OLR1 were measured by RTqPCR. *p < 0.05 versus miR-3571 NC. No significance (N.S.) p>0.05 versus miR-3571 NC. **D.** Relative luciferase activity were measured with a luciferase reporter assay. *p < 0.05 versus CLDN1 3'UTR-WT. E. Expression of CLDN1 in the aortas of SHRs and WKYs were measured by RTqPCR and Western blotting. *p < 0.05 versus WKYs. F. Expression of Claudin 1 in VSMCs subjected to 5% or 15% CS for 12h and 24h were measured by Western blot analysis. *p < 0.05 versus 5% CS. Data represent means ± SD and at least three separate experiments. Statistical comparisons were conducted using a Student's t test.

Using bioinformatics methodology (Targetscan), it was found that CLDN1 and oxidized low density lipoprotein receptor 1(OLR1) are potential target genes for miR-3571 (Figure 2A). To explore the effect of miR3571 on the expression of CLDN1 and OLR1 in VSMCs, miR3571 mimic was transfected into cells. As shown in Figure 2B, the expression of miR3571 increased significantly in the mimic group in comparison with the NC group. The mRNA expression of CLDN1 and OLR1 in VSMCs transfected with miR-3571 mimic or NC was then measured. As displayed in Figure 2C, the miR-3571 mimic significantly decreased the expression of CLDN1 but did not affect the expression of OLR1, indicating that CLDN1 may be a target of miR-3571. What's more, miR-3571 antagomir significantly increased the mRNA and protein level of CLDN1 in VSMCs which further support that CLDN1 may be a target of miR-3571 (Supplementary Figure S5). Therefore, a dual-luciferase reporter assay was performed to confirm the binding of the target. As presented in Figure 2D, cotransfection with miR3571 mimic and CLDN1 3'UTRWT significantly decreased luciferase activity, but no significant change was observed in the CLDN1 3'UTRMUT and miR3571 mimic co-transfection group. In addition, the expression of CLDN1 was higher in the aortic tissue of SHRs than in WKYs, as observed using RT-qPCR and Western blot analysis (Figure 2E). Furthermore, the protein expression levels of CLDN1 increased in VSMCs when subjected to 15% CS compared with 5% CS (Figure 2F). Together, these data suggest that CLDN1 was a downstream target of miR3571 and may be involved in the progression of hypertension.

### 3.4 MiR-3571 inhibited VSMC proliferation

**Figure 3:**
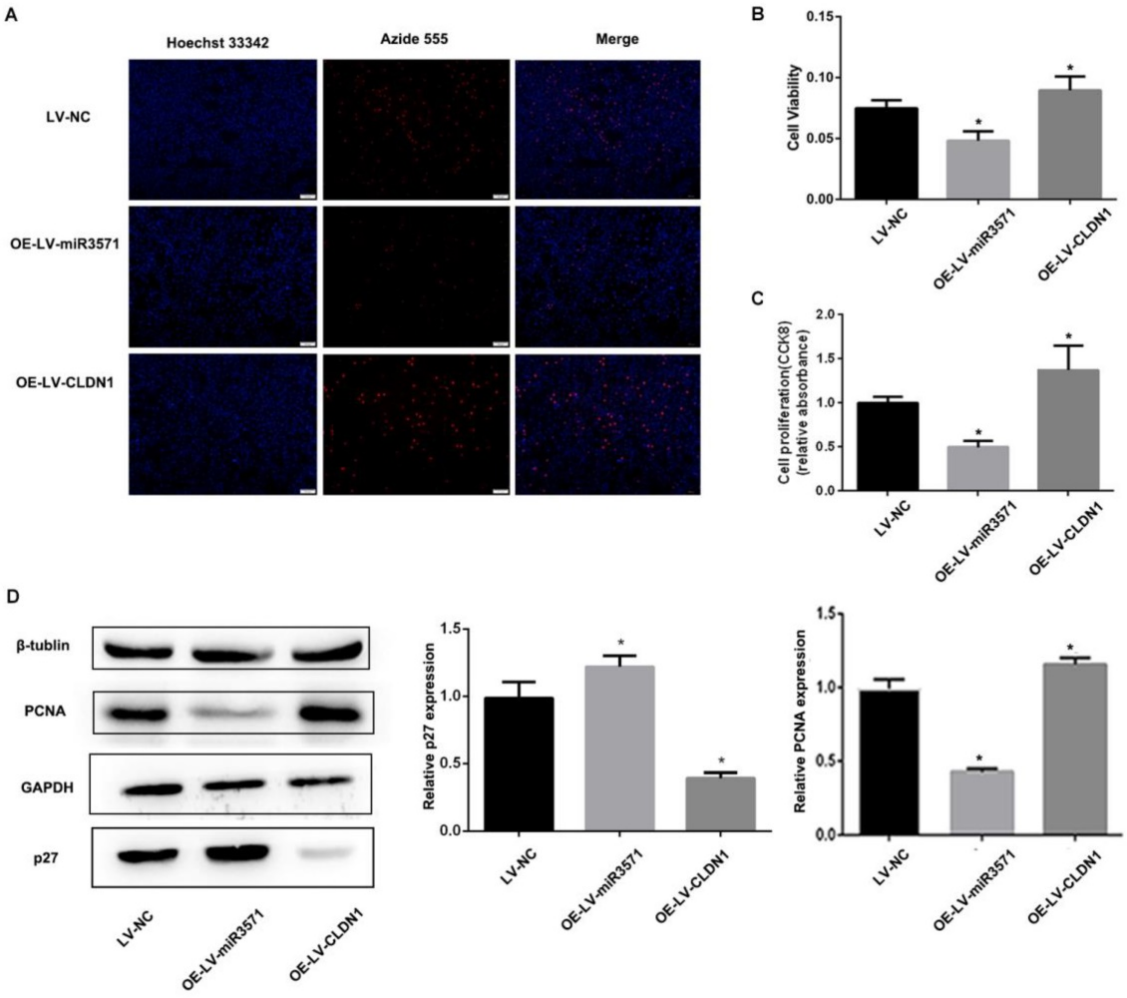
** MiR3571 overexpression inhibited proliferation of VSMCs. A.** Proliferation of VSMCs was assessed by EdU assay and cell proliferation quantification. *p < 0.05 versus LV-NC. **B.** Proliferation of VSMCs was assessed by CCK8 assay. *p < 0.05 versus LV-NC. **C.** Expression of PCNA and p27 were measured by Western blot analysis. *p < 0.05 versus LV-NC. Data represent means ± SD in three separate experiments. Significance was determined by oneway ANOVA with Tukey's multiple comparisons test.

**Figure 4:**
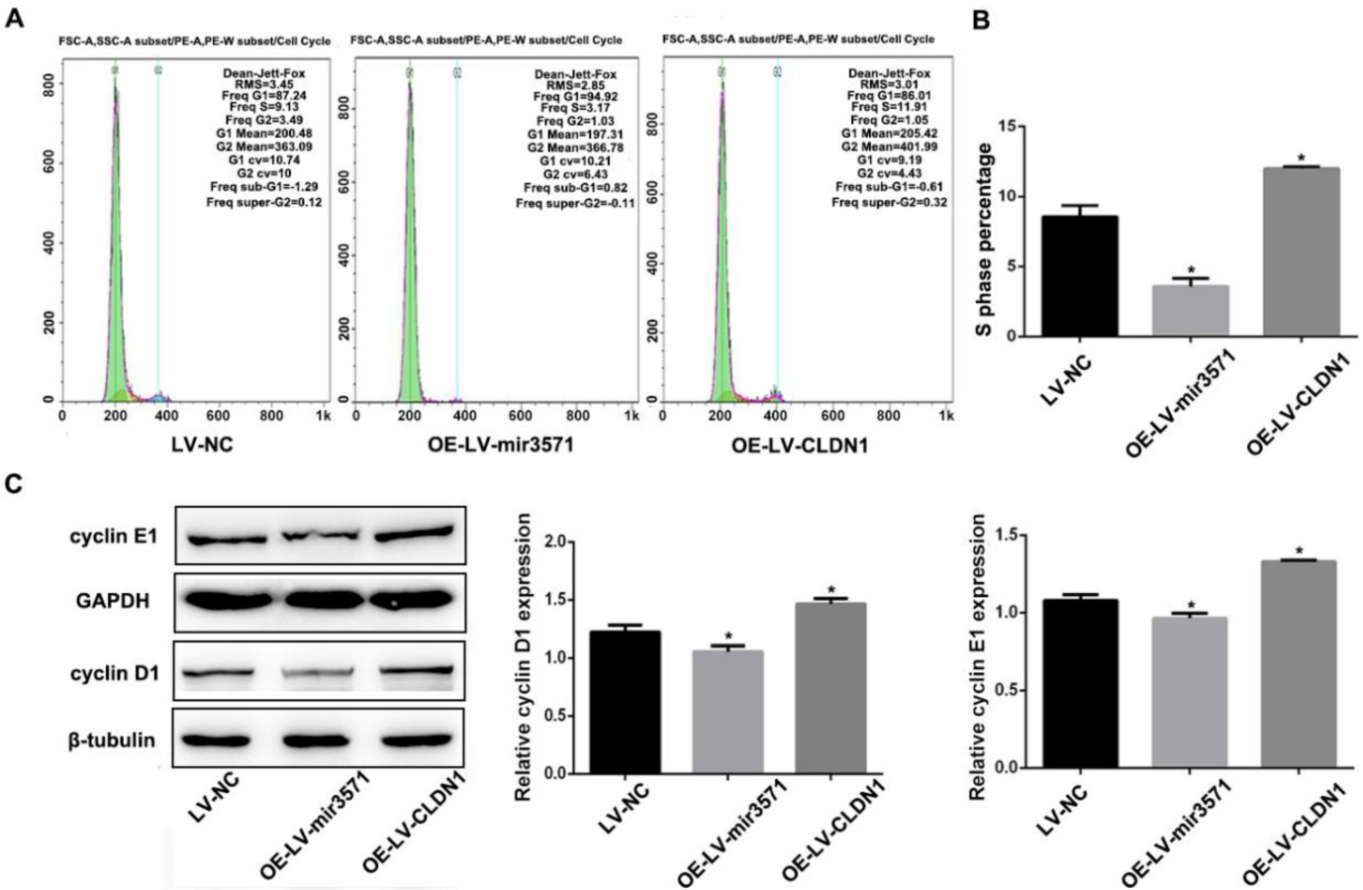
** MiR3571 overexpression affected cell cycle of VSMCs. A.** Representative DNA histograms of propidium iodide fluorescence in cells, as assessed by flow cytometry. **B.** Percentage of cells in the S phase of the cell cycle was quantified by flow cytometric analysis. **C.** Expression of cyclin D1 and E1 were measured by Western blot analysis. *p< 0.05 versus LV-NC.

To evaluate the effects of miR-3571 and CLDN1 on the regulation of VSMC function, VSMC cell lines stably overexpressing CLDN1 and miR-3571 were established and verified by RT-qPCR and Western blot analysis (Supplementary Figure S2).The proliferation of the different stable VSMC cell lines was then measured with EdU and CCK8 assays. We found that the proliferation of VSMCs was slower upon overexpressing miR-3571 than that of VSMCs transfected with LV-NC, while the proliferation of VSMCs with CLDN1 overexpression was higher than that of control VSMCs(LV-NC) (Figures 3A, B, and C). In addition, the overexpression of miR-3571 significantly increased the expression of p27 but decreased the levels of proliferating cell nuclear antigen (PCNA). By contrast, CLDN1 upregulation significantly reduced the p27 levels but increased the expression of PCNA (Figure 3D). To investigate how miR-3571 and CLDN1 regulated VSMC proliferation, we further analyzed cell cycle progression by flowcytometry (Figure 4A). We found that the percentage of cells in S phase was significantly lower upon enforced expression of miR-3571, while the overexpression of CLDN1 appeared to have the opposite effects (Figure 4B). Consistent with these results, miR-3571 decreased the levels of cyclins D1 and E1, while CLDN1 overexpression increased their expression (Figure 4C). Taken together; these results indicate that miR-3571 regulates VSMC proliferation through CLDN1.

### 3.5 Role of miR-3571 and CLDN1 in VSMC migration

**Figure 5:**
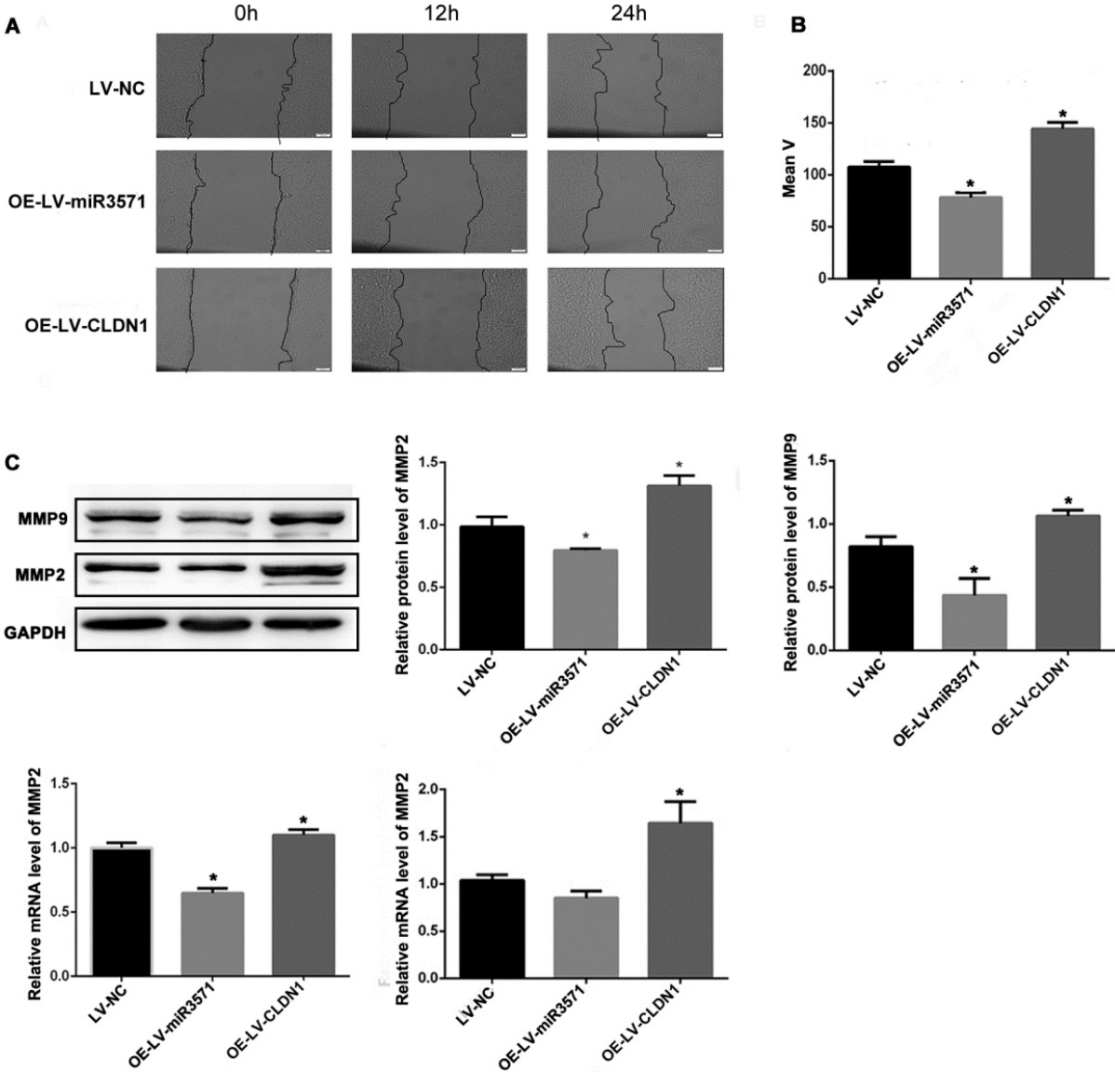
** MiR3571 overexpression inhibited migration of VSMCs. A.** Migration of VSMCs was assessed by wound healing assay. **B.** Cell migration rate quantification. *p < 0.05 versus LV-NC.** C.** mRNA and protein expression levels of MMP2 and MMP9 were measured by RT-qPCR and Western blot analysis. *p < 0.05 versus LV-NC. Data represent means ± SD in three separate experiments. Significance was determined by oneway ANOVA with Tukey's multiple comparisons test.

As shown in Figures 5A and B, a wound-healing assay was used to evaluate the effect of miR-3571 and CLDN1 on the migration of VSMC. The migration of VSMCs was decreased upon overexpressing miR-3571 compared with that of VSMCs transfected with LV-NC while the migration of VSMCs with CLDN1 overexpression was increased in comparison with that control VSMCs (LV-NC). In addition, the expression of migrationrelated proteins, as measured by Western blotting and RT-qPCR, indicated that miR-3571 upregulation reduced the expression of matrix metalloproteinase 2 (MMP2) and matrix metalloproteinase 9 (MMP9) at both the mRNA and protein levels and CLDN1 overexpression upregulated the expression of MMP2 and MMP9 at both the mRNA and protein levels (Figure 5C). Together, these observations reveal that miR-3571 overexpression suppress the migration of VSMCs via the targeting of CLDN1.

### 3.6 Role of miR-3571 and CLDN1 in the phenotypic modulation of VSMCs

**Figure 6:**
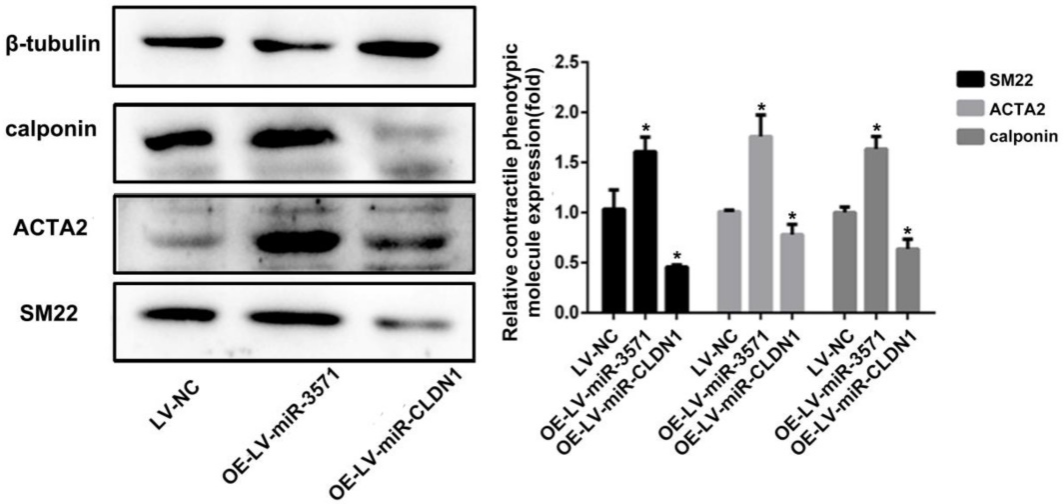
** MiR-3571 overexpression inhibited the transformation of VSMCs from a contractile to proliferating phenotype.** The expression of three contractile protein, SM22, ACTA2 and calponin, were measured by Western blot analysis. *p < 0.05 versus LV-NC. Data represent means ± SD in three separate experiments. Significance was determined by oneway ANOVA with Tukey's multiple comparisons test.

VSMCs can transit from quiescent, differentiated cells to proliferating, migrating cells, a process known as phenotypic modulation. Smooth muscle 22α protein (SM22), ACTA2, and calponin are common contractile proteins used as markers of differentiated smooth muscle cells. As shown in Figure 6, miR-3571 overexpression significantly increased the expression of SM22, ACTA2, and calponin and therefore inhibited the transformation of VSMCs from a contractile to proliferating phenotype. Additionally, CLDN1 upregulation significantly decreased the expression of SM22, ACTA2, and calponin. Taken together, these results indicate that miR-3571 inhibit the transformation of VSMCs from contractile to proliferating phenotype through the suppression of CLDN1.

### 3.7 The effect of miR-3571 or CLDN1 inhibition on VSMCs function

The above results illustrate that miR-3571 or CLDN1 overexpression significantly affect the phenotypic modulation, proliferation and migration of VSMCs. We also explored the effects of miR-3571 or CLDN1 inhibition on phenotypic modulation, proliferation and migration of VSMCs. As shown in Supplementary Figure S6, when miR-3571 was inhibited with the treatment of miR-3571 antagomir, the expression of SM22 and ACTA2 significantly down-regulated while the level of MMP9 and PCNA significantly increased, which illustrated that the inhibition of miR-3571 induced the transformation of VSMCs from a contractile to proliferating phenotype and induced the proliferation and migration of VSMCs. On the other hand, when CLDN1 was inhibited with the treatment of CLDN1 siRNA, the expression of SM22 and ACTA2 significantly up-regulated while the level of MMP9 and PCNA significantly decreased, this illustrated that the inhibition of CLDN1 inhibited the transformation of VSMCs from a contractile to proliferating phenotype and inhibited the proliferation and migration of VSMCs. These results further corroborated that the correlation between miR-3571 and CLDN1 expression levels dictated the response of SMCs in culture.

### 3.8 CLDN1 affects cell function through the regulation of extracellular signal-regulated kinase (ERK) phosphorylation

**Figure 7:**
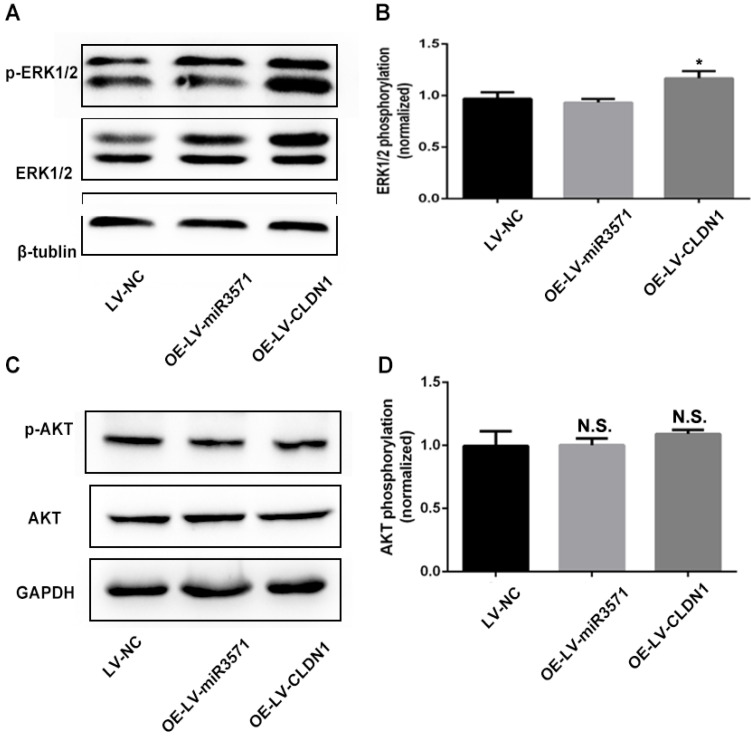
** CLDN1 overexpression activated ERK1/2. A** and **B.** Protein expression levels of total ERK and p-ERK were measured by Western blot analysis. *p < 0.05 versus LV-NC. **C** and **D.** Protein expression levels of total AKT and p-AKT were measured by Western blot analysis. No significance (N.S.) p>0.05 versus LV-NC. Data represent means ± SD in three separate experiments. Significance was determined by oneway ANOVA with Tukey's multiple comparisons test.

The results above suggest that miR-3571 regulate the proliferation, migration, and phenotypic modulation of VSMCs through CLDN1, but its underlying mechanism remains unknown. The ERK and protein kinase B (AKT) signaling pathways are known to participate in the regulation of VSMC proliferation, migration, and phenotypic modulation. Therefore, ERK1/2 and AKT activity was examined using Western blot analysis. The results indicate that the phosphorylation of ERK1/2 increased substantially due to the overexpression of CLDN1 while the overexpression of miR-3571 had no impact on the phosphorylation of ERK1/2 (Figures 7A and B). However, neither the overexpression of miR-3571 nor CLDN1 affected the phosphorylation of AKT (Figures 7C and D). These data indicate that CLDN1 promotes the proliferation and migration of VSMCs through the activation of ERK1/2.

## Discussion

As is generally accepted, hypertension is a major factor leading to mortality and the single most important predisposing contributor to cardiovascular disease 23, 24. Multiple studies have reported that miRNA-based post-transcription modification mechanisms significantly affect the progression of hypertension 11, 12, 22. It's still worth exploring how miRNA expression is regulated in this pathological process. Hydroxymethylation of DNA is a newly found epigenetic modification and reported to regulate gene expression. Therefore, we intend to explore DNA hydroxymethylation's role in miRNA regulation in hypertension. In the present study, we initially explored hydroxymethyl cytosine DNA immunoprecipitation (hMeDIP) sequencing in the thoracic aortas of SHRs and WKYs and found 5 miRNAs with differentially hydroxymethylated promoter regions. We then verified the level of hydroxymethylation in the promoter region of miR-3571 and its expression in the aortic tissue of SHRs and WKYs, and then finally investigated the effects of miR3571 on the proliferation, migration and phenotypic modulation of VSMCs and their possible regulatory mechanisms. We found that the level of hydroxymethylation in the promoter region and the expression of miR-3571 was downregulated in the aortas of SHRs compared with WKYs, and that miR-3571 inhibited VSMC proliferation, migration and phenotypic modulation from contractile to proliferating phenotype by targeting CLDN1.

Recently, DNA hydroxymethylation has been a focus of investigation because of its potential function in the regulation of gene expression, especially hydroxymethylation of the gene promoter regions 25, 26. We found 5 miRNAs whose hydroxymethylated regions displayed significant differential expression in SHRs compared with WKYs and confirmed with glucosylation-coupled hydroxymethylation-sensitive qPCR that there was less promoter hydroxymethylation of miR-3571 in cells of the thoracic aorta in SHRs compared with WKYs. More detailed studies demonstrated that the expression of miR-3571 also decreased in the thoracic aortas of SHRs compared with WKYs, supported by RT-qPCR data. In addition, miR-3571 was also down-regulated in VSMCs subjected to pathological hypertensive CS (15%) compared with physiological levels (5%) using a Flexcell® FX-5000™ tension system. Bioinformatics analysis and experimental validation revealed that CLDN1 was a downstream target of miR-3571, results that were supported by a dual-luciferase reporter assay, RT-qPCR, and Western blot analysis. CLDN1 is a critical structural and functional component of tight junctions (TJs) and shown to be a hallmark of multiple pathological conditions 27. Currently, the principal research focus of CLDN1 is in cancer studies, including tumorigenesis and epithelial-mesenchymal transition, and it has been reported to be principally associated with the regulation of cellular functions such as cell proliferation, migration, and invasion 28-31. Its role in hypertension has rarely been reported. A recent study did report that CLDN1 may be involved in the pathogenesis of PAH 32. This suggests that miR-3571 and CLDN1 may be involved in the progression of hypertension.

Mounting evidence supports the hypothesis that the development of hypertension accompanies aberrant and excessive proliferation and migration of VSMCs 33. It has been demonstrated that numerous miRNAs regulate VSMC proliferation and migration through numerous mechanisms 10-12, 22. In the present study, LV-NC, OE-LV-miR3571, and OE-LV-CLDN1 vectors were transfected into established stable VSMC cell lines. The results of the EdU and CCK8 assays indicated that miR3571 overexpression inhibited the capability of VSMCs to proliferate and that CLDN1 overexpression induced VSMCs to proliferate. Concurrently, miR3571 overexpression diminished the expression of the proliferative phenotype protein marker PCNA combined with increased p27 expression, while CLDN1 upregulation suppressed the expression of p27 and increased PCNA expression. The results of cell cycle analysis indicated similar trends. Additionally, strong inhibition of VSMC migration coupled with decreased active MMP2 and MMP9 expression was observed with OE-LV-miR-3571, the converse of that observed with OE-LV-CLDN1. These findings confirm that miR3571 overexpression inhibits proliferation and migration of VSMCs, at least partly through CLDN1.

Previous reports have already demonstrated that phenotypic modulation of VSMCs participates in the progression of hypertension 6, 34. The phenotypic modulation of VSMCs from contractile to those that are proliferating is accompanied by lower levels of contractile protein expression and higher levels of extracellular matrix and inflammatory cytokine expression. The process serves as a major factor that initiates vascular remodeling in a number of cardiovascular diseases, such as atherosclerosis, hypertension, vascular stenosis, and diabetic vascular complications 35, 36. A recent review concluded that miR-221, miR-146a, miR-24, and miR-26a are involved in the phenotypic modulation of VSMCs 8. In the present study, we investigated the effects of miR-3571/CLDN1 on the phenotypic modulation of VSMCs. The expression of contractile proteins was examined using Western blot analysis. We found that the expression of contractile proteins was up-regulated with OE-LV-miR-3571 but down-regulated with OE-LV-CLDN1. The results suggest that miR-3571 inhibits the transformation of VSMCs from a contractile to proliferating phenotype and that CLDN1 induced the proliferating phenotype.

Although the effect of miR-3571 and CLDN1 on the function of VSMCs was demonstrated in detail through different experimental approaches, the specific mechanisms require further investigation. To clarify the underlying mechanism of miR-3571 and its downstream target CLDN1, we attempted to investigate the associated signaling pathways. ERK1/2 is a key subfamily of mitogen-activated protein kinases (MAPKs) and controls a broad range of cellular activities and physiological processes, including proliferation, differentiation, migration, and apoptosis. A recent study stated that CLDN1 regulates pulmonary artery smooth muscle cell proliferation through the activation of ERK1/2 32. Therefore in the present study, we examined the levels of total ERK1/2 and phosphorylated ERK1/2 through Western blot analysis to verify the activity of ERK1/2. The results revealed that CLDN1 overexpression increased the activity of ERK1/2 while miR-3571 did not.

## Conclusions

In summary, we have established that the expression levels of miR-3571 decreased in the thoracic aortic tissue of SHRs compared with WKYs accompanied with the reduced levels of hydroxymethylation within the promoter region of miR-3571. We have presented evidence that miR3571 regulates the proliferation, migration, and phenotypic modulation of VSMCs through the targeting of CLDN1. These findings reveal that miR-3571 is involved in the progression of hypertension and representing a potential approach to the treatment of hypertension and other related cardiovascular diseases.

## Supplementary Material

Supplementary figures and table.

## Figures and Tables

**Table 1:** Specific primer sequences for Glucosylation-Coupled Methylation-Sensitivity qPCR

Name	Sequences,5'-3'	Tm(℃)
5-hmc-mir-3571-F	GGAATAGGGCTGTTAG	48
5-hmc-mir-3571-R	GTAATGTCCGCCAAGA	48

**Table 2:** Gene-specific primer sequences for RT-qPCR.

Name	Primers sequences, 5'-3'	Tm(℃)
MMP2	F CTGGGTTTACCCCCTGATGTCC R AACCGGGGTCCATTTTCTTCTTT	61.85 56.64
MMP9	F GGGACGCAGACATCGTCATC R TCGTCATCGTCGAAATGGGC	58.65 58.36
GAPDH	F AGCTTCCCATTCTCAGCCTTGACT R ACAAGATGGTGAAGGTCGGTGTGA	60.4 60.4
CLDN1	F AGGCAACCAGAGCCTTGATGGTAA R CATGCACTTCATGCCAATGGTGGA	60.80 60.57
OLR1	F GCTATCCTTTCTTGGGTGTAAAAC R TTGCTTCTGGTCTTTGTCTCTG	53.57 54.74
miR-3571-RT	GTCGTATCCAGTGCAGGGTCCGAGGTATTCGCACTGGATACGACTATGGA	72.65
U6-RT	GTCGTATCCAGTGCAGGGTCCGAGGTATTCGCACTGGATACGACAAAATA	73.4
miR-3571-F	CGCGCGTACACACTTCTTTACAT	57.81
U6-F	AGAGAAGATTAGCATGGCCCCTG	60.2
All-R	AGTGCAGGGTCCGAGGTAT	62

**Table 3:** The primer sequences of CLDN1 3'UTR-WT and CLDN1 3'UTR-MUT

Name	Sequences,5'-3'
CLDN1 3'UTR-WT	Forward AACGAGCTCGCTAGCCTCGAGGGGTGCTCCTTAAGTGTGTA Reverse CCTGCAGGTCGACTCTAGACGTGTGGGAAAGGTCAGTGGA
CLDN1 3'UTR-MUT	Forward AACGAGCTCGCTAGCCTCGAGGGGTGCTCCTTACTCTGAGC Reverse CCTGCAGGTCGACTCTAGACGTGTGGGAAAGGTCAGTGGA

**Table 4:** The miRNAs whose hydroxymethylated regions displayed significant differential expression in SHRs compared with WKYs.

RefSeq_name	GeneSymbol	chr	tss	tts	strand	Genomic_position
NR_037349	Mir3065	chr10	108793392	108793495	+	chr10:108794261-108794460
NR_031783	Mir338	chr10	108793475	108793409	-	chr10:108794261-108794460
NR_037360	Mir3571	chr18	2087900	2088011	+	chr18:2087581-2087800
NR_032116	Mir1	chr18	2087998	2087911	-	chr18:2087581-2087800
NR_031777	Mir330	chr1	81358759	81358856	+	chr1:81357121-81357320

RefSeq_name: Refseq accession number of the differentially hydroxymethylated region associated gene. Symbol: Gene symbol of the differentially hydroxymethylated region associated gene. Chr: The chromosome of the differentially hydroxymethylated region associated gene. TSS: The start sites of the differentially hydroxymethylated region associated gene. TTS: The end sites of the differentially hydroxymethylated region associated gene. Strand: The strand of the differentially hydroxymethylated region associated gene. Genomic_position: Chr, Start and End of the differentially hydroxymethylated region.

**Table 5:** The sequence of miR-3571 antagomir and *CLDN1* siRNA

Name	5'-3' sequence	3'-5' sequence
miR-3571 antagomir	UAUGGAAUGUAAAGAAGUGUGUA	
*CLDN1* siRNA	CCAGAGCCUUGAUGGUAAUTT	AUUACCAUCAAGGCUCUGGTT

## Abbreviations

SHRs: spontaneously hypertensive rats
WKYs: normotensive Wistar-Kyoto rats
hMeDIP: hydroxymethylcytosine DNA immunoprecipitation
VSMCs: vascular smooth muscle cells
CLDN1: claudin1
CS: cyclic strain
SMCs: smooth muscle cells
miRNAs: micro-RNAs
DNMTs: DNA methyltransferases
5-mC: 5-methylcytosine
TET: Ten-eleven translocation
5-hmC: 5-hydroxymethyl-cytosine
MYH11: smooth muscle-myosin heavy chain
ACTA2: smooth muscle actin
PAH: pulmonary arterial hypertension
SDRs: Sprague-Dawley rats
DhMRs: Differentially hydromethylated regions
DMEM: Dulbecco's modified Eagle medium
SMA: smooth muscle α-actin
NC: negative control
HEK: human embryonic kidney
CCK8: cell counting kit-8
OLR1: oxidized low density lipoprotein receptor 1
PCNA: proliferating cell nuclear antigen
MMP2: matrix metalloproteinase 2
MMP9: matrix metalloproteinase 9
SM22: Smooth muscle 22α
ERK: extracellular signal-regulated kinase
AKT: protein kinase B
MAPKs: mitogen-activated protein kinases
