# Supplementary material for: MiR-3571 modulates the proliferation and migration of vascular smooth muscle cells by targeting claudin 1
Source: Int J Med Sci. 2022 Mar 6;19(3):511–24. doi: 10.7150/ijms.64639 (PMC8964332; doi:10.7150/ijms.64639)
Supplement: Supplementary file 1 — Supplementary figures and table. [file ijmsv19p0511s1.pdf]

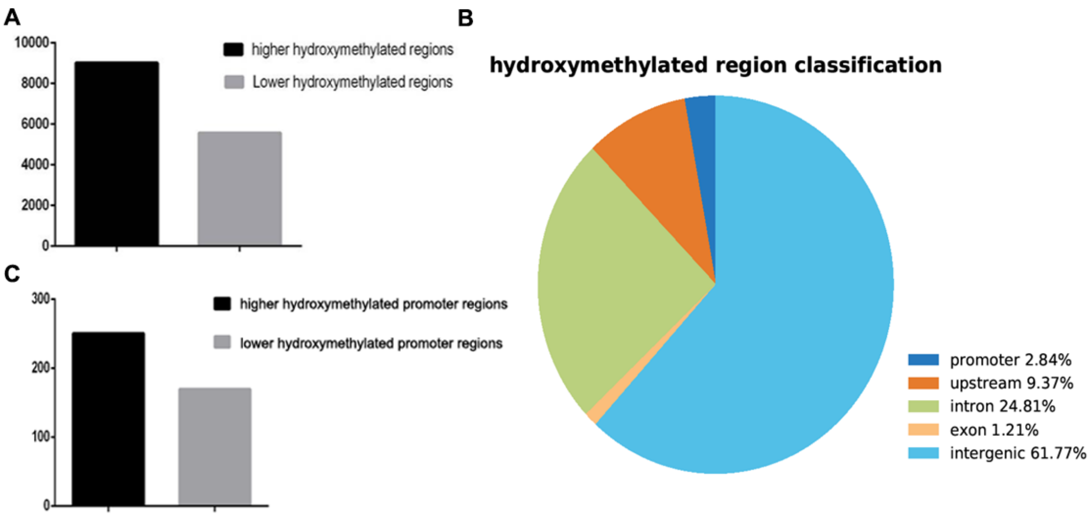

Supplement figure.S1. Differentially hydroxymethylated regions in SHRs compared with WKYs as identified by hMeDIP-Seq (n=3). A. Bar chart of differentially hydroxymethylated regions. B. Hydroxymethylated region classification. C. Bar chart of differentially hydroxymethylated promoter regions.

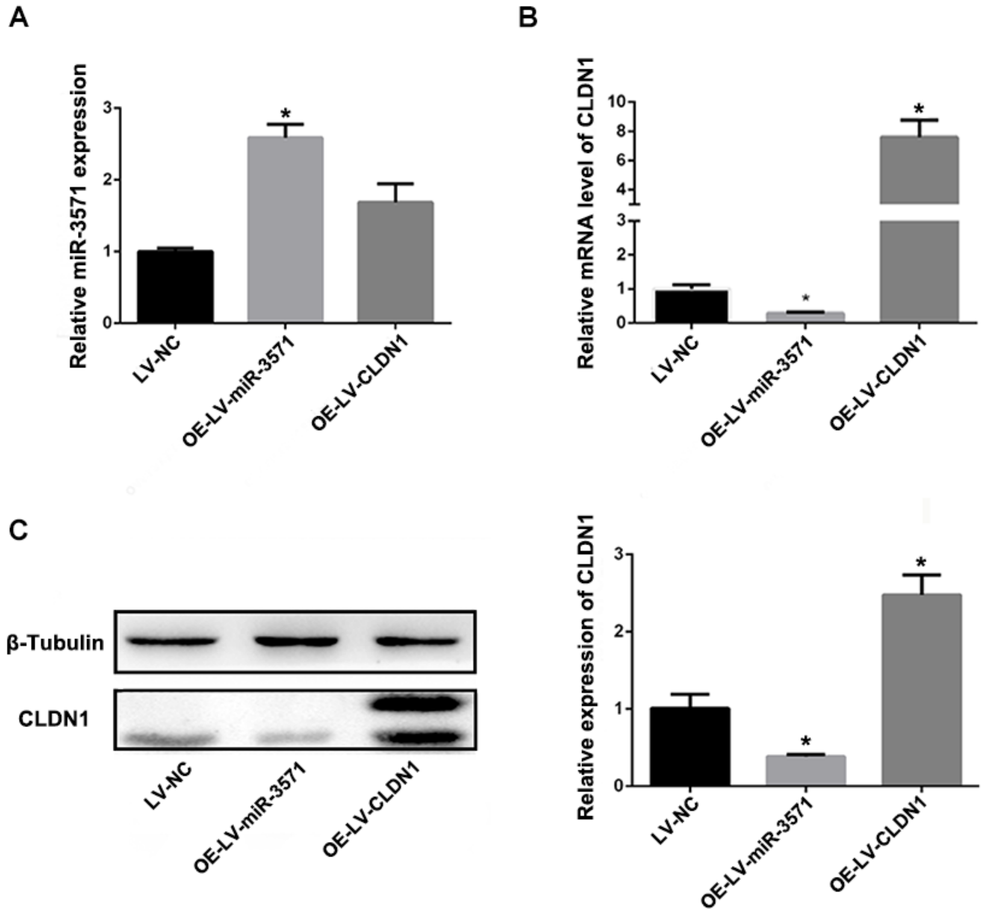

Supplement figure.S2. CLDN1 and miR-3571 overexpression in stable VSMC cell lines was established. A. Expression of miR-3571 in three stable cell lines was confirmed by RT-qPCR. *p

< 0.05 versus LV-NC. B. mRNA levels of CLDN1 in three stable cell lines measured by RT-qPCR. *p < 0.05 versus LV-NC. C. Protein expression levels of CLDN1 in three stable cell lines measured by Western blot analysis. *p < 0.05 versus LV-NC. Data represent means ± SD in at least three separate experiments. Significance was determined by one-way ANOVA with Tukey’s multiple comparisons test.

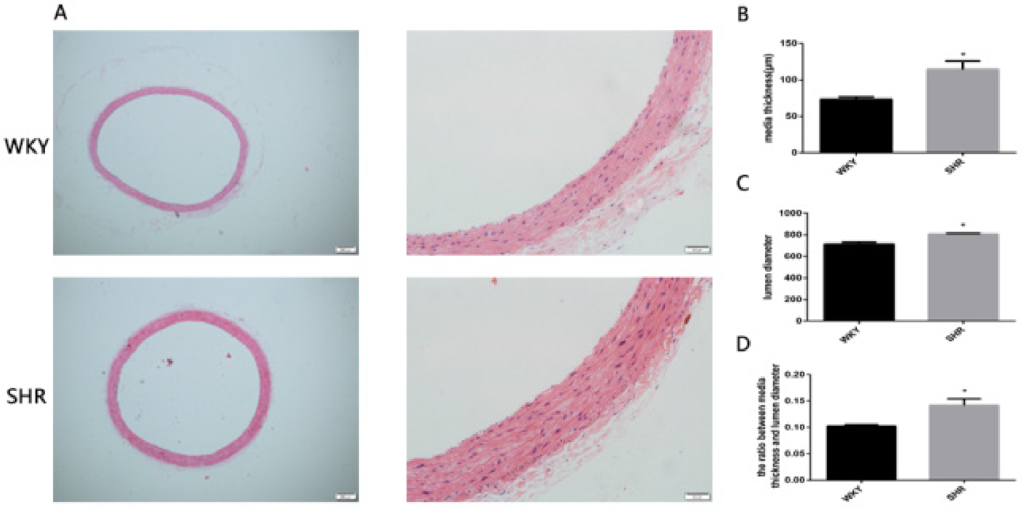

Supplement figure.S3. Vascular remodeling is present in the thoracic aorta of SHRs. A. Representative HE stain image of WKYs and SHRs thoracic aorta. Left panel: Scale bar: 200 μm. Right panel: Scale bar: 50 μm. B. increased media thickness in the thoracic aorta of SHRs compared to WKYs. C. increased lumen diameter in the thoracic aorta of SHRs compared to WKYs. D. increased ratio between media thickness and lumen diameter in the thoracic aorta of SHRs compared to WKYs .*p< 0.05 represents statistical significance compared to WKYs.

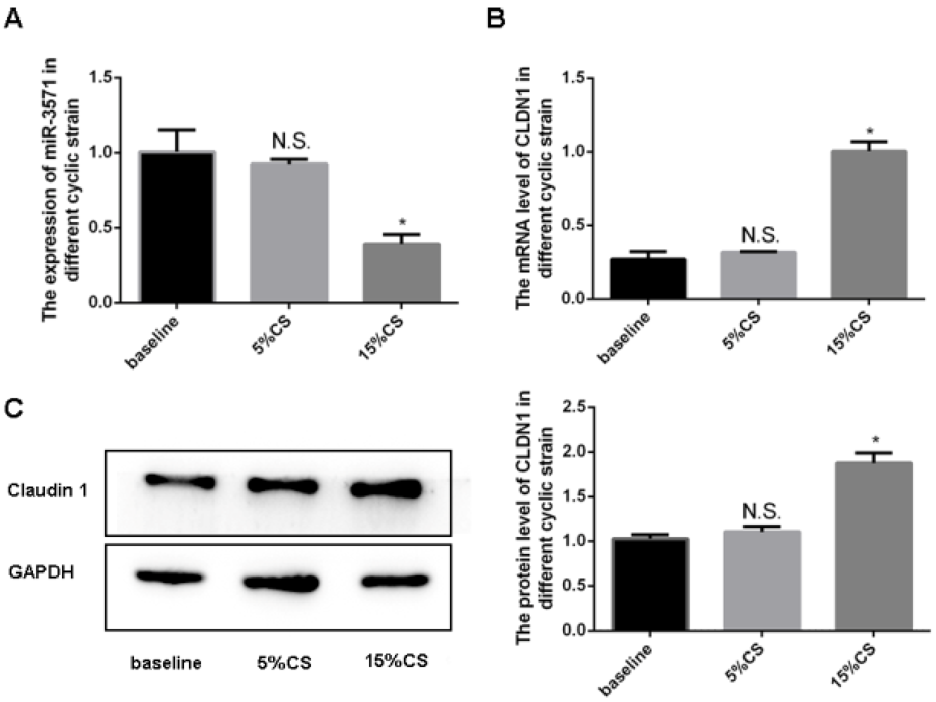

Supplement figure.S4. The effect of different mechanical stress on miR-3571 and CLDN1. A. Expression of miR-3571 in different cyclic strain was confirmed by RT-qPCR. $*p < 0.05$ versus baseline. N.S. $p>0.05$ versus baseline. B. mRNA levels of CLDN1 in different cyclic strain was confirmed by RT-qPCR. $*p < 0.05$ versus baseline. N.S. $p>0.05$ versus baseline. C. Protein expression levels of CLDN1 in different cyclic strain was confirmed by western blot. $*p < 0.05$ versus baseline. N.S. $p>0.05$ versus baseline. Data represent means ± SD in at least three separate experiments. Significance was determined by one-way ANOVA with Tukey's multiple comparisons test.

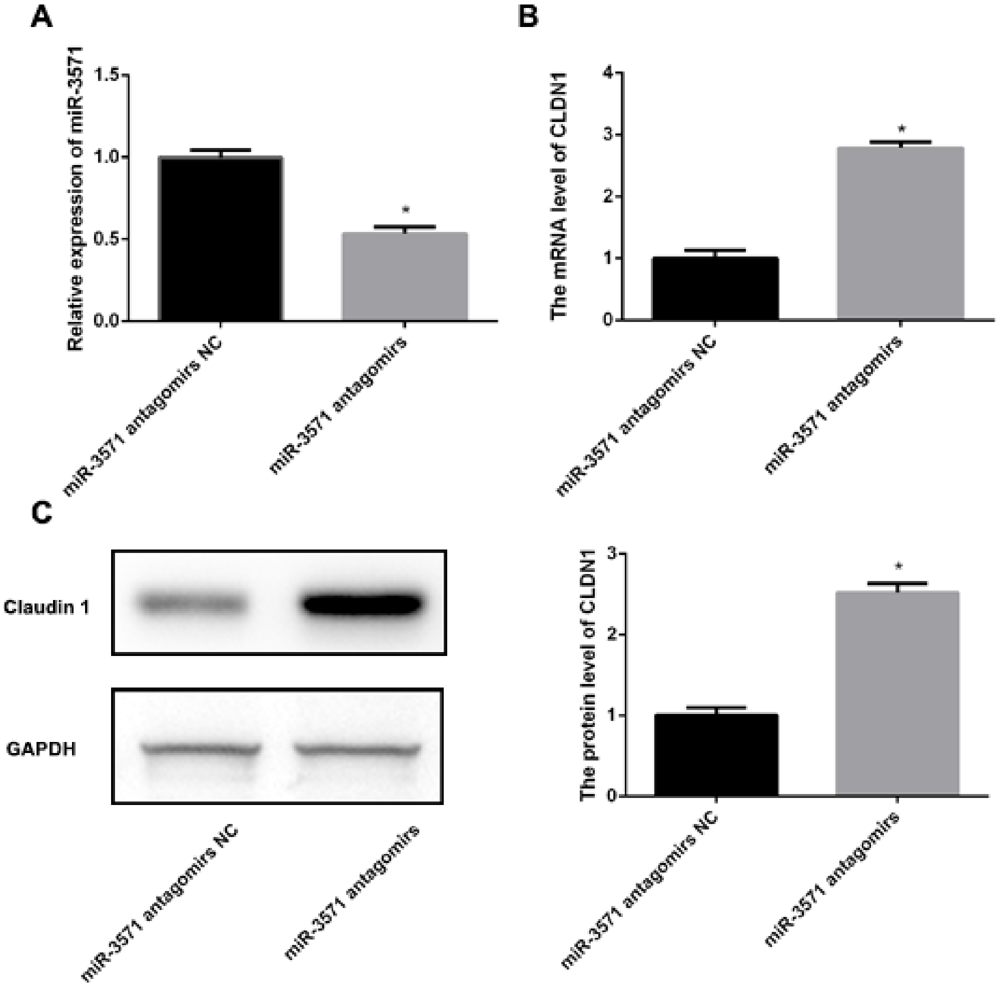

Supplement figure.S5. miR-3571 antagomirs increased the expression of CLDN1. A. Expression of miR-3571 after transfection with miR-3571 antagomirs was measured by RT-qPCR. *$p < 0.05$ versus miR-3571 antagomirs NC. B. Expression of CLDN1 were measured by RT-qPCR. *$p < 0.05$ versus miR-3571 antagomirs NC. C. Protein expression levels of CLDN1 was confirmed by western blot. *$p < 0.05$ versus miR-3571 antagomirs NC. Data represent means ± SD and at least three separate experiments. Statistical comparisons were conducted using a Student's t test.

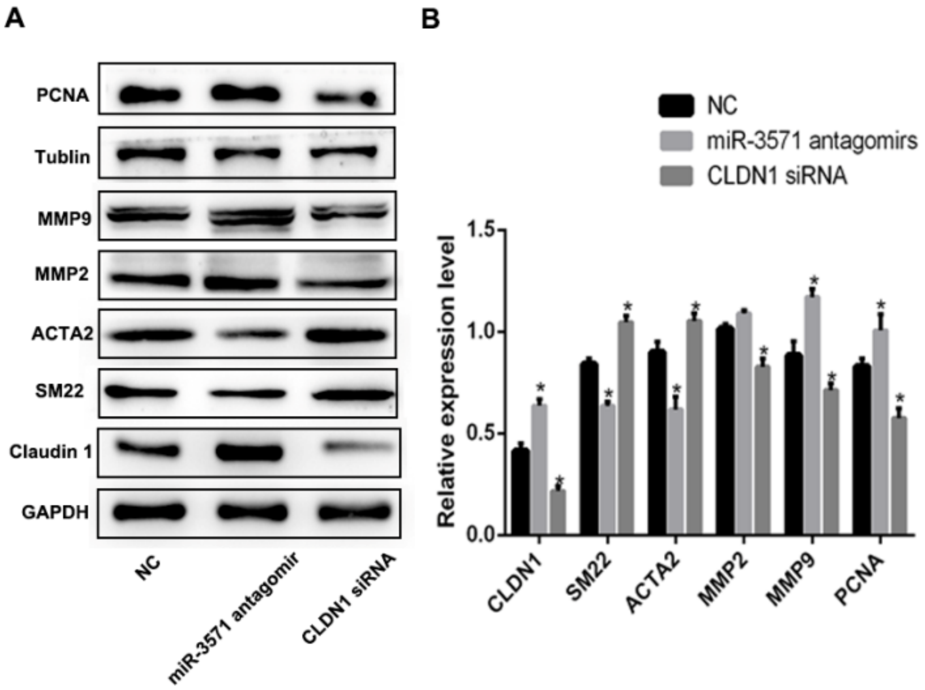

Supplement figure.S6. The protein level of CLDN1,SM22,ACTA2,MMP2,MMP9,PCNA in VSMCs treat with miR-3571 antagomir or CLDN1 siRNA. A. Western blot results for CLDN1, SM22, ACTA2, MMP2, MMP9, PCNA . B The bar chart of western blot results for these protein . *$p < 0.05$ versus NC. Data represent means ± SD in three separate experiments. Significance was determined by one-way ANOVA with Tukey's multiple comparisons test.

Tables

Supplement table1. The heart rate, systolic blood pressure, mean blood pressure, diastolic blood pressure of the male WKYs and SHRs (n=3,aging 18 weeks) which were used to perform Hydroxymethylcytosine DNA immunoprecipitation (hMeDIP) sequencing.

	Heart rate	Systolic blood pressure	Mean blood pressure	Diastolic blood pressure
WKY1	481	129	109	98
WKY2	464	133	112	101
WKY3	447	128	101	88
SHR1	478	192	164	149
SHR2	445	201	166	149
SHR3	499	185	147	128
